# PSMA PET/CT–Targeted Biopsy in Men with Negative or Equivocal Multiparametric MRI and Exploratory Dynamic Total-Body PET: The FUPERMAN Study

**DOI:** 10.2967/jnumed.126.272108

**Published:** 2026-09

**Authors:** Andrea Farolfi, Caterina Maria Paola Sgro, Irene Brusa, Daniele Dall’Olio, Francesco Romei, Massimiliano Presutti, Matteo Droghetti, Andrea Di Giorgio, Riccardo Mei, Beniamino Corcioni, Caterina Gaudiano, Cristina Mosconi, Riccardo Schiavina, Paolo Castellucci, Stefano Fanti, Lorenzo Bianchi

**Affiliations:** 1 Nuclear Medicine, IRCCS Azienda Ospedaliero-Universitaria di Bologna, Bologna, Italy;; 2 Department of Medical and Surgical Sciences, University of Bologna, Bologna, Italy;; 3 Department of Urology, IRCCS Azienda Ospedaliero-Universitaria di Bologna, Bologna, Italy;; 4 Nuclear Medicine, Alma Mater Studiorum, University of Bologna, Bologna, Italy; and; 5 Department of Radiology, IRCCS Azienda Ospedaliero-Universitaria di Bologna, Bologna, Italy

**Keywords:** prostate cancer, PSMA PET/CT, targeted biopsy, mpMRI-negative, total-body PET

## Abstract

Prostate-specific membrane antigen (PSMA) PET/CT may enhance detection of clinically significant prostate cancer (csPCa) in men with negative or equivocal multiparametric MRI (mpMRI), a setting where diagnostic uncertainty persists. This study assessed the diagnostic performance of PSMA PET/CT–guided targeted biopsy (PET-TB) versus systematic biopsy (SB) and explored the value of semiquantitative (SUV_max_) and dynamic total-body PET parameters in predicting csPCa. **Methods:** This is a prospective, single-center study that enrolled 63 men with PI-RADS 2–3 mpMRI findings between March 2023 and July 2025. All underwent [^68^Ga]Ga-PSMA-11 PET/CT followed by transperineal prostate biopsy. A subset of 11 patients underwent dynamic total-body PET. The primary endpoint was detection of csPCa (International Society of Urological Pathology score of ≥2). We compared diagnostic yields of PET-TB and SB, evaluated predictors via logistic regression, and analyzed dynamic PET data using kinetic modeling. **Results:** PET-TB detected significantly more csPCa than did SB (37% vs. 21%; *P* = 0.021), achieving a sensitivity of 96%, a specificity of 49%, a positive predictive value of 57%, and a negative predictive value of 95%. Combining PET-TB with SB increased the csPCa detection rate to 41%. SUV_max_ independently predicted csPCa (odds ratio, 51.2; 95% CI, 5.6–2713.9; *P* = 0.015), with a threshold of 11.4 yielding 100% specificity. In dynamic PET analysis, csPCa showed irreversible kinetics, and *K_i_*/*k*_3_ parameters improved lesion discrimination beyond SUV, especially in equivocal (PRIMARY score 3) lesions. **Conclusion:** PSMA PET/CT–guided targeted biopsy enhances csPCa detection in men with negative or equivocal mpMRI, with added predictive value from SUV_max_ and dynamic PET metrics. These promising findings should be validated in larger multicenter trials.

## Visual Abstract

**Figure:**
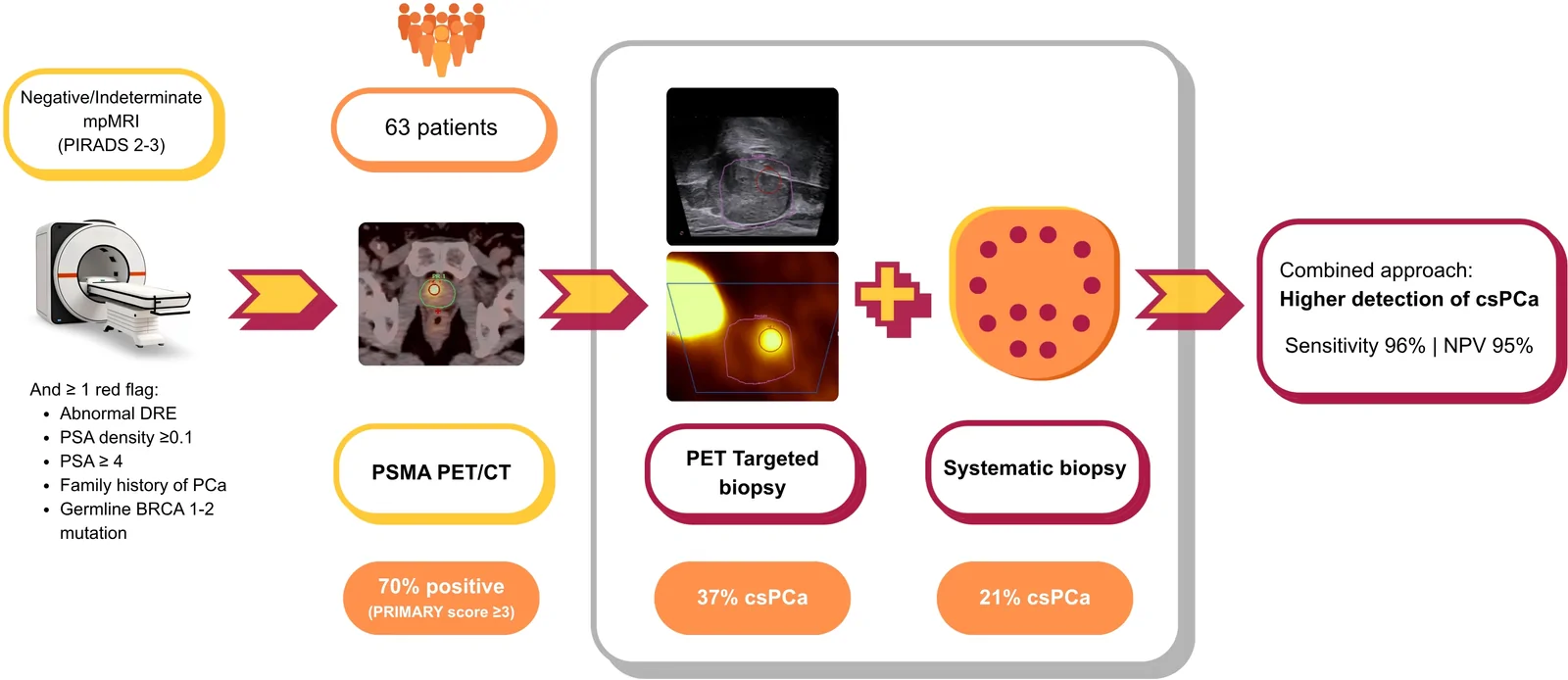

Prostate cancer (PCa) diagnosis typically involves prostate biopsy using a systematic or image-guided approach. Multiparametric MRI (mpMRI) is now considered the cornerstone for detecting and localizing clinically significant prostate cancer (csPCa), enabling targeted biopsy and reducing overdiagnosis of indolent disease (1,2). However, its diagnostic accuracy can be suboptimal in certain scenarios. Up to 20%–30% of csPCa cases may be missed in patients with negative or equivocal mpMRI findings (PI-RADS 1–3), especially when small, anterior, or low-volume tumors are present (1,3). Moreover, cribriform and intraductal patterns are mostly invisible with mpMRI, and some MRI-invisible lesions had high Decipher score, underlining potentially aggressive disease due to mutation in genes involved in cancer progression (4). In such patients, the diagnostic approach remains undefined, and repeat systematic biopsies (SBs) are frequently performed despite their modest yield and potential morbidity.

Prostate-specific membrane antigen (PSMA)–targeted PET has recently emerged as a promising diagnostic tool for primary PCa detection (5). Beyond its established role in staging and assessing recurrence, PSMA PET offers high tumor-to-background contrast within the prostate, allowing for precise localization of intraprostatic lesions. Several prospective and retrospective studies, including the PRIMARY study, have demonstrated that combining PSMA PET with mpMRI enhances sensitivity and negative predictive value (NPV) for detecting csPCa compared with mpMRI alone, with potential to reduce unnecessary biopsies (5,6). Moreover, when integrated into image fusion platforms, PSMA PET enables targeted biopsy of PET-positive lesions (PSMA PET–targeted biopsy [PET-TB]), providing high detection rates in either biopsy-naïve men or those with previous negative biopsy results. However, the feasibility and diagnostic role of real-time software-based PSMA PET fusion biopsies for detecting csPCa remain underexplored in the literature.

Evidence for PSMA PET is particularly scarce in men with negative or equivocal mpMRI—a population representing a critical diagnostic challenge. A negative mpMRI may prompt biopsy deferral, at the risk of missing csPCa, whereas PI-RADS 3 lesions frequently trigger SB with limited yield. Determining whether PSMA PET can refine risk stratification and guide targeted sampling in this setting could have a direct impact on clinical decision-making.

The advent of next-generation PET/CT scanners, including long–axial-field-of-view and total-body PET/CT, has further advanced the field of molecular imaging (7,8). These systems offer superior signal collection efficiency and reduced noise, leading to improved lesion conspicuity over conventional scanners (9–11). Importantly, they also enable total-body dynamic imaging and multiparametric quantification beyond conventional SUV metrics (12). Indeed, static PET metrics offer only a snapshot of tracer distribution over a limited time interval, reflecting both specific and nonspecific signals. In contrast, dynamic PET coupled with pharmacokinetic modeling may provide absolute, time-resolved measures of tracer behavior, uncovering biologically meaningful patterns invisible to conventional imaging (13). Exploratory studies have shown that kinetic profiling refines lesion characterization and may provide novel diagnostic insights in intermediate- to high-risk PCa (14–16). Such an approach may prove particularly impactful even in patients with low-risk PCa and in equivocal cases, where conventional SUV-based analysis falls short.

In this prospective single-center study named fusion PSMA PET in MRI-negative men (FUPERMAN), we aimed to investigate the diagnostic performance of PSMA PET–guided biopsy in men with negative (PI-RADS 2) or equivocal (PI-RADS 3) mpMRI findings. We compared PET-TB with SB, assessed the added value of combining both approaches, and explored the potential role of quantitative PET parameters, including SUV_max_ and, in a subgroup, dynamic total-body PET metrics, in predicting csPCa.

## MATERIALS AND METHODS

### Study Design and Population

FUPERMAN is an open-label, single-center, nonrandomized, prospective study. The study cohort comprised 63 consecutive patients who underwent PET fusion biopsy at our institution between March 2023 and July 2025. mpMRI was performed in each patient before prostate biopsy, and only patients with negative mpMRI or equivocal (i.e., PI-RADS 2 or 3) were selected. Prostate-specific antigen (PSA) was reassessed at least 4–6 mo after mpMRI to confirm persistently abnormal values before PSMA PET and biopsy. The inclusion criteria were as follows: abnormal digital rectal examination, a PSA density of 0.1 ng/mL^2^ or higher, a PSA level of 4 ng/mL or higher, a family history of PCa, or known germline BRCA 1–2 mutation. Patients included in the study underwent PSMA PET for diagnostic purposes within 12 mo of previous mpMRI, followed by a transperineal prostate biopsy within 6 mo after the PSMA PET scan. This study was approved by our institutional board (Ethical Committee AVEC of Bologna, approval 244/2016/O/Oss). All participants gave written informed consent (when applicable) in accordance with the Declaration of Helsinki to collect their data.

### mpMRI Imaging

All MRI examinations were performed with a 3.0-T MR scanner (Ingenia Omega HP; Philips Healthcare). The protocol study of the prostate was performed according to the European Society of Urogenital Radiology guidelines (17). All MR images were scored using PI-RADS version 2.1 by 2 genitourinary radiologists with more than 10 y of experience in prostate MRI (18).

### PSMA PET Imaging

PSMA PET was performed 60 min after injection of a total dose of a 1.8–2.2 MBq/kg of [^68^Ga]Ga-PSMA-11 (according to the European Association of Nuclear Medicine procedure guidelines) using a diagnostic-quality non–contrast-enhanced CT (modulated mAs, 9–240; 100–120 kV) (19). PSMA PET examinations were performed with a GE Discovery MI scanner (GE HealthCare) and a United Imaging uEXPLORER total body scanner (United Imaging Healthcare). PSMA PET images were acquired starting from feet to vertex. Subsequently, a delayed scan of the pelvis was acquired at 90-min postinjection after voiding. Each scan was reviewed by 2 experienced nuclear medicine physicians with at least 8 y of experience with PSMA ligands, using the PRIMARY score for reporting (20). All intraprostatic PSMA PET findings were considered negative for csPCa in the case of PRIMARY scores 1–2 but were considered positive for csPCa in the case of lesions with PRIMARY scores 3–5. PSMA PET was reported according to PROMISE version 2 criteria (21).

### Dynamic Total-Body PSMA PET

This subanalysis included 11 patients who underwent 60-min dynamic PET acquisition on a total-body system (field of view, 194 cm; uEXPLORER) immediately after intravenous administration of [^68^Ga]Ga-PSMA-11. Low-dose CT scans (72 mAs, 120 kV) were acquired before the dynamic PET scan for attenuation correction and anatomic localization. Dynamic PET images were acquired in list mode, corrected for radioactive decay, scatter, attenuation, and random and were reconstructed with an image matrix of 150 × 150 pixels, a slice thickness of 4 mm, using a 3-dimensional ordered-subset expectation maximization algorithm with 4 iterations and 20 subsets and incorporating time-of-flight. Dynamic PET data were reconstructed into a total of 66 dynamic frames: 30 × 2 s, 12 × 10 s, 6 × 30 s, 12 × 120 s, 6 × 300 s. Volumes of interest (VOIs) were manually delineated by 2 experienced nuclear medicine physicians on focal intraprostatic areas with increased uptake (SUV_mean_, >3.5) and on reference prostate tissue without focal significant uptake (SUV_mean_, <3.5) on the summed image frames from the last 5 min of the dynamic scan. The 3.5 threshold was based on institutional experience and SUV_mean_ distribution of histologically negative reference prostate tissue within the dynamic subgroup. The obtained VOIs were imported to the 0–60 dynamic scan and manually repositioned frame per frame to compensate for possible patient repositioning. Accuracy of PET VOI placement in reference prostate and lesions was confirmed with postbiopsy pathology specimens.

### PSMA PET Fusion–Target Biopsy

Prostate biopsies were performed via transperineal approach with “free-hand technique” in an outpatient setting under local anesthesia (10 mg of lidocaine plus 1 mg of mepivacaine) by 1 expert urologist, using UroFusion software and Esaote MyLaB X9 platform, as previously described (22). All participants underwent transperineal SB with a minimum of 8 cores according to prostate volume. In the same session, men with negative mpMRI (PI-RADS 2) and positive PSMA PET (i.e., PRIMARY scores 3, 4, or 5) underwent additional real-time software-based fusion PSMA–target biopsy with at least 3 cores for each suspicious area. To perform PSMA-target biopsy, prostate volume contours on CT and PSMA-positive lesions (regions of interest) were manually delineated by 1 nuclear medicine physician and 1 urologist. UroFusion software consists of a rigid navigation system: prostate contour previously acquired on PSMA PET imaging is automatically fused in real-time with a dynamic overlay of prostate contour at transrectal ultrasound. Thus, PSMA PET–derived regions of interest are automatically overlapped in real-time on prostate 3-dimensional volume at ultrasound, and the spatial location of each biopsy cores site was recorded in real-time (Supplemental Fig. 1; supplemental materials are available at http://jnm.snmjournals.org). Patients with equivocal findings on mpMRI (PI-RADS 3) underwent real-time systematic prostate biopsy, as well as a PSMA PET/CT–targeted biopsy and an MRI-targeted biopsy.

### Pharmacokinetic Modeling

Time–activity curves were extracted for each VOI over the full dynamic [^68^Ga]Ga-PSMA-11 acquisition. Image-derived input function was assessed using a linear segment of the descending aorta automatically delineated with TotalSegmentator (23). A Patlak plot and a relative equilibrium plot were performed to investigate tracer uptake via multitime graphical analysis using frames acquired for 10 min or greater to minimize the influence of early nonequilibrium. Data were standardized, and weighted linear regression was used to estimate the net-influx rate (*K_i_*) for Patlak and distribution volume for relative equilibrium. Kinetic modeling was conducted for 2-tissue compartment model scenarios to account for reversible and irreversible uptakes. The Levenberg–Marquardt algorithm was used to estimate *K*_1_, *k*_2_, *k*_3_, *k*_4_ (when applicable), blood fraction, and time delay of image-derived input function. *K_i_* and distribution volume were also calculated when feasible. Model adequacy was compared using Akaike information criterion and mean absolute scalar error; when the Vuong test indicated a significant difference (*P* < 0.05), the model with lower Akaike information criterion was selected; otherwise, the more parsimonious model was preferred. A schematic representation of the 2-tissue compartment model is provided to facilitate interpretation of kinetic parameters (Supplemental Fig. 2).

### Statistical Analysis

Baseline patient characteristics and PET/CT parameters were summarized as counts and percentages, median and interquartile range, or mean and SD. When appropriate, continuous PET values were dichotomized to create scientifically appropriate groups.

Continuous variables, such as SUV_max_, were compared between patients with and without csPCa using the nonparametric Mann–Whitney test because of the asymmetric distribution of the variables. SUV_max_ was analyzed against the International Society of Urological Pathology (ISUP) grade group categories using the Wilcoxon statistical test. The CIs were estimated using the Clopper–Pearson exact method. Kinetic parameters were compared between lesions and reference prostate using the Wilcoxon test (2-sided, α = 0.05). Associations among compartmental parameters (e.g., *K_i_*, *K*_1_, *k*_2_, *k*_3_) and SUV metrics were assessed with Spearman rank correlations. All statistical tests were performed using SPSS 22.0 (IBM).

## RESULTS

### Patient Characteristics

**FIGURE 1.**
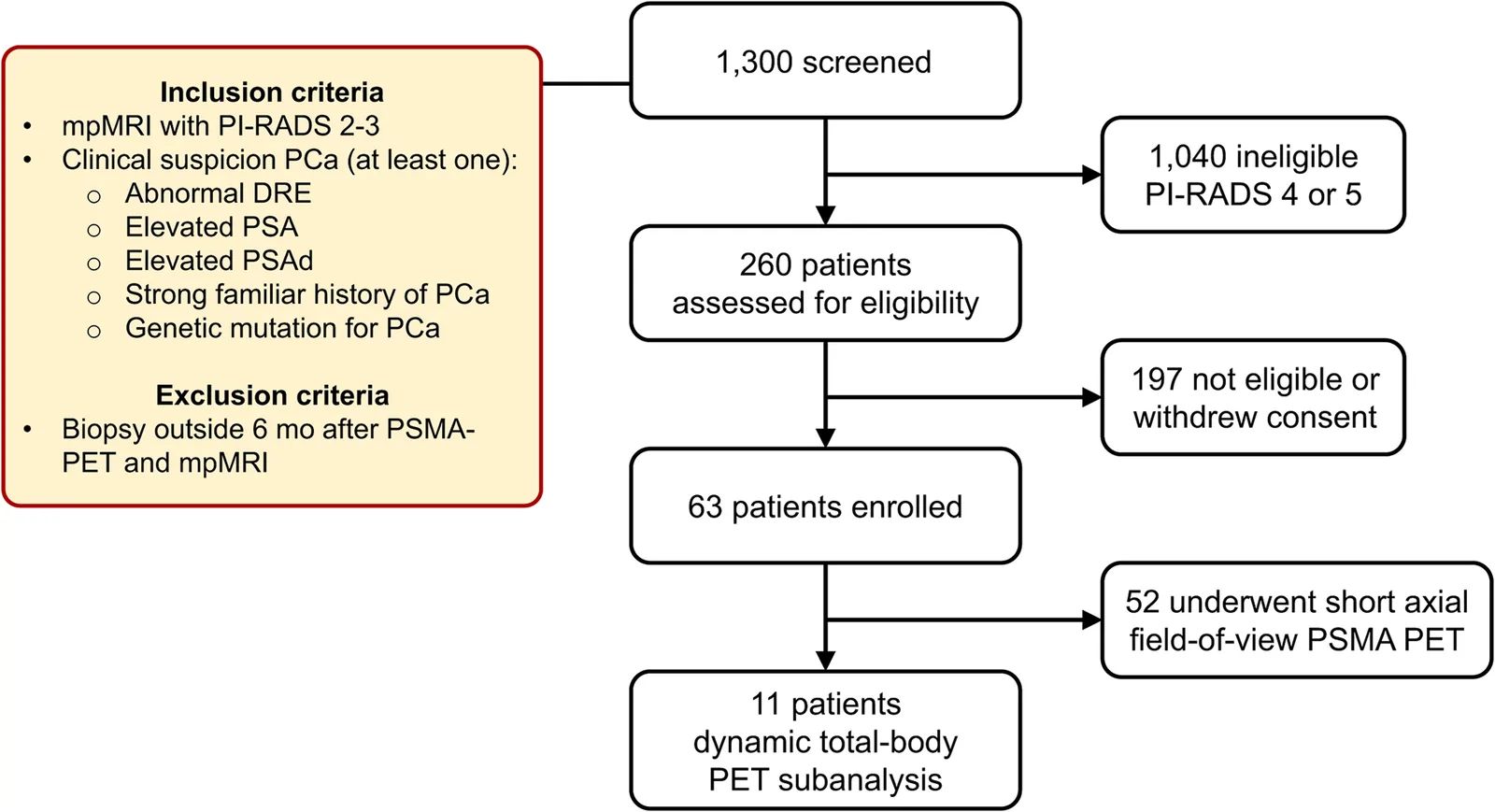
Consort diagram for patient selection. DRE = digital rectal exam; PSAd = PSA density.

Sixty-three men underwent mpMRI, PSMA PET, and biopsy (Fig. 1). Patient characteristics are detailed in Table 1. Overall, 26 of the 63 (41%) harbored csPCa (ISUP ≥2) and 44 of the 63 (70%) had a positive PSMA PET (PRIMARY ≥3) (Table 2; Supplemental Table 1). Of the men, 21 of 51 (41%) with PI-RADS 2 and 5 of 12 (42%) with PI-RADS 3 had csPCa on biopsy. Overall, 4 of the 63 men (6%) had PSMA-positive pelvic lymph nodes or metastatic disease on PSMA PET.

**TABLE 1.** Patient Characteristics (*n* = 63)

Parameter	Median	*n*
Age at biopsy (y)	67 (58–70)	
Latest PSA (ng/mL)	12.8 (7.4–17.7)	
Clinical setting		
Biopsy naïve		40 (64)
Previous negative biopsy		17 (27)
Previous ISUP 1 (active surveillance)		6 (9)
Time from MRI to biopsy (mo)	8 (4–12)	
Time from PSMA PET to biopsy (d)	27 (14–38)	
PSA density (ng/mL^2^)	0.23 (0.17–0.34)	
MRI prostate volume (cm^3^)	50 (31–71)	
PI-RADS		
2		51 (81)
3		12 (19)
SUV_max_ (local PSMA PET read)		
At 60 min	5.7 (3.8–11.0)	
At 90 min	5.8 (3.6–13.2)	
PRIMARY score		
1		5 (8)
2		14 (22)
3		18 (29)
4		11 (18)
5		15 (24)
PSMA PET		
Negative (PRIMARY <3)		19 (30)
Positive (PRIMARY ≥3)		44 (70)
Number of biopsy cores	13 (10–15)	
ISUP grade group		
No cancer		22 (35)
1		15 (24)
2		10 (16)
3		8 (13)
4		6 (9)
5		2 (3)

Values in parentheses are interquartile range for median and percentage for *n*.

**TABLE 2.** Overall and csPCa Detection According to PRIMARY Score

PRIMARY score	*n*	Median SUV_max_	Overall PCa	csPCa	ISUP ≥4
1	5 (8)	2.4 (2.3–2.8)	2 (40)	0 (0)	0 (0)
2	14 (22)	3.7 (3.2–4.3)	3 (21)	1 (7)	0 (0)
3	18 (29)	6.0 (4.4–6.8)	16 (89)	6 (33)	0 (0)
4	11 (17)	6.8 (4.8–7.7)	5 (45)	4 (36)	1 (10)
5	15 (24)	26.6 (16.1–38.7)	15 (100)	15 (100)	7 (47)

Qualitative data are number and percentage. Continuous data are median and interquartile range.

### Diagnostic Performance

**FIGURE 2.**
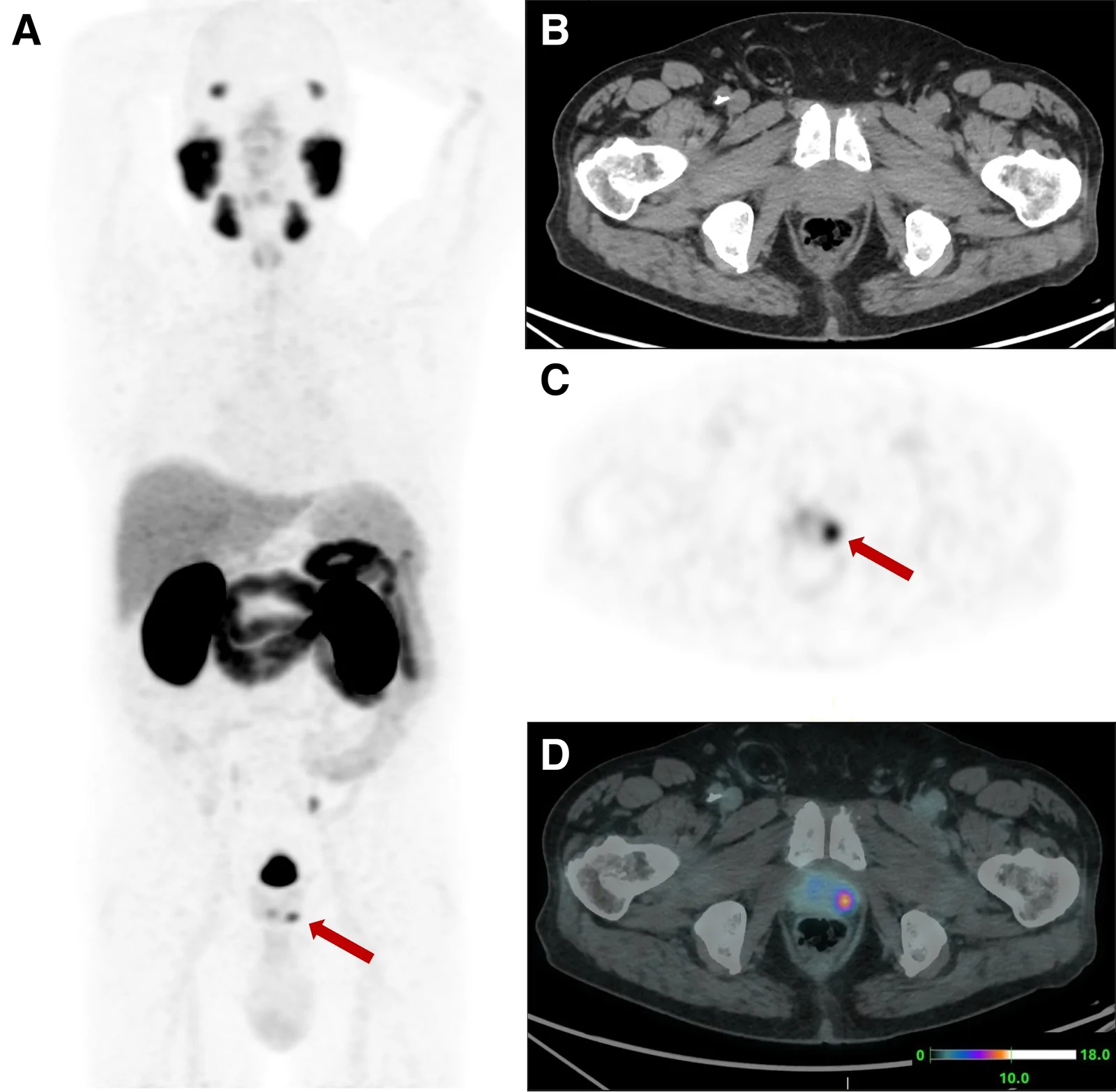
Upstage with PET-guided biopsy. 70-y-old patient with suspicious PCa (PI-RADS 2; initial PSA, 15.9; PSA density, 0.4). Exam showed intense focal [^68^Ga]Ga-PSMA-11 uptake (SUV_max_, 9.5; PRIMARY score 4) in left peripheral zone at midlevel shown maximum-intensity projection (A), transaxial view of CT image (B), PET image (C), and transaxial fused PET/CT image (D). Transperineal PSMA PET Targeted prostate biopsy revealed csPCa, ISUP 5, whereas SB revealed csPCa, ISUP 2.

**FIGURE 3.**
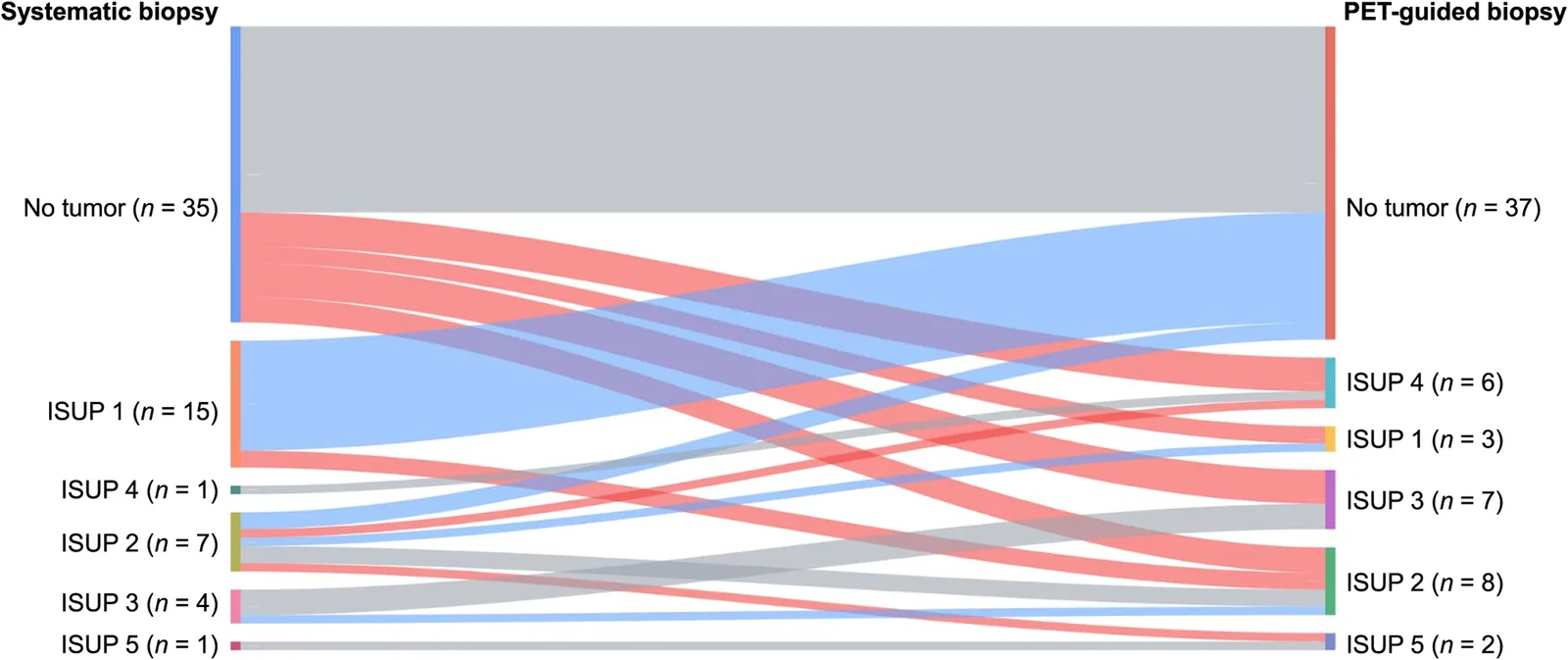
Sankey diagram showing ISUP grade migration between systematic and PSMA PET–guided biopsies. Red lines indicate same patient upstaging, and blue lines indicate downstaging between biopsy approaches.

PSMA PET fusion–targeted biopsy alone detected significantly more csPCa than SB alone (37% vs. 21%; *P* = 0.021). Sensitivity, specificity, positive predictive value, and NPV for PSMA PET fusion–target biopsy alone were 96%, 49%, 57%, and 95%, respectively (Table 3). When PSMA PET fusion–target biopsy was combined with SB, the csPCa detection rate increased to 26 of 63 patients (41%) (Table 4). PSMA PET correctly identified 25 of 26 (96%) csPCa cases. Agreement between PSMA fusion and SB for the detection of csPCa patients was poor (κ = 0.116, *P* = 0.349), and PSMA PET revealed a higher ISUP grade group compared with SB in 17 of 63 patients (27%) (Figs. 2 and 3). In 3 patients, csPCa was missed by PSMA PET fusion–target biopsy; in 2 patients, ISUP grade group 2 was detected by SB but was not identified on PSMA PET. In 1 patient, SB revealed ISUP grade group 2 csPCa, whereas PSMA PET was positive (PRIMARY score 4), but the corresponding PSMA PET–guided fusion biopsy was negative for clinically significant disease.

**TABLE 3.** Diagnostic Test Parameters

	PSMA fusion alone		PSMA fusion with SB	
Parameter	Negative	Positive	Negative	Positive
No clinically significant cancer	19	21	18	19
csPCa	0	23	1	25
Sensitivity (%)	96 (80–100)			
Specificity (%)	49 (32–66)			
PPV (%)	57 (49–65)			
NPV (%)	95 (72–99)			

Qualitative data are number. Values in parentheses are 95% CI.

PPV = positive predictive value.

**TABLE 4.** Detection Rate

Parameter	PSMA fusion alone	SB alone	PSMA fusion with SB
PRIMARY score 1–2*			
Overall PCa	—	5 (26)	5 (26)
csPCa	—	1 (5)	1 (5)
PRIMARY score 3, 4, or 5^†^			
Overall PCa	26 (59)	23 (52)	35 (80)
csPCa	23 (52)	12 (27)	25 (57)

* *n* = 19

† *n* = 44.

Values in parentheses are percentage.

### PSMA PET Parameters

On quantitative analysis, the receiver-operating-characteristic curve for SUV_max_ had an area under the curve (AUC) value of 0.847 (95% CI, 0.750–0.945), higher than the AUC for PSA density (0.698; 95% CI, 0.564–0.832). The combined model yielded the highest AUC (0.849; 95% CI, 0.749–0.950) (Supplemental Fig. 3). An SUV_max_ threshold of 11.4 achieved 100% specificity and 58% sensitivity for csPCa. PSMA intensity (SUV_max_) was associated with csPCa (*P* < 0.001) (Table 5), and SUV_max_ increased significantly across ISUP grade groups (*P* < 0.001). Median PSMA SUV_max_ in men without csPCa on biopsy was 4.3 (95% CI, 4.1–5.7) versus 14.8 (95% CI, 12.6–26.2) for ISUP 2–5 malignancy (Supplemental Table 2). In our cohort, patients also underwent a delayed scan at 90 min postinjection. An increase in SUV_max_ between 60 and 90 min had a higher rate of csPCa compared with those with stable or decreasing SUV_max_ (49% vs. 26%); this corresponded to an odds ratio (OR) of 2.69 (95% CI, 0.88–8.27), but the difference did not reach statistical significance (*P* = 0.08).

**TABLE 5.** Univariate Logistic Regression Analysis for the Prediction of csPCa

Parameter	β	OR	95% CI	*P*
Age (y)	0.05	1.06	0.99–1.13	0.092
Previous biopsy	0.73	2.068	0.700–6.111	0.189
PSA (ng/mL)	0.00	1.00	0.92–1.07	0.900
PSA density (ng/mL^3^)	0.66	1.93	1.24–3.41	0.010
Prostate volume (cm^3^)	−0.04	0.96	0.93–0.98	0.004
mpMRI result*	0.02	1.02	0.27–3.64	0.975
SUV_max_	0.32	1.37	1.16–1.75	0.002
PSMA-PET result^†^	3.16	23.68	4.30–444.96	0.003

* PI-RADS 2 vs. 3

† PRIMARY score 1–2 vs. 3, 4, or 5.

### Clinical, Laboratory, and Radiologic Characteristics

The presence of csPCa did not differ between patients with PI-RADS 2 and PI-RADS 3 lesions (*P* = 0.975) (Supplemental Table 3). On univariate analysis, a significantly higher risk of csPCa was associated with an increase in PSA density, SUV_max_, PRIMARY score, and a decrease in prostate volume (all *P* < 0.05) (Table 5). On multivariable analysis combining clinical, laboratory, and mpMRI/PET data, independent factors associated with the presence of csPCa were age (OR 2.98; 95% CI, 1.27–9.21; *P* < 0.05), prostate volume (OR, 0.12; 95% CI, 0.02–0.44; *P* < 0.05), and SUV_max_ (OR, 51.2; 95% CI, 5.57–2713.98; *P* < 0.05) (Table 6).

**TABLE 6.** Multivariate Logistic Regression Analysis for the Prediction of csPCa

Parameter	β	OR	95% CI	*P*
Age (y)	1.09	2.98	1.27–9.21	0.025
Prostate volume (cm^3^)	−2.12	0.12	0.02–0.44	0.007
SUV_max_	3.94	51.23	5.57–2713.98	0.015

### Dynamic Total-Body PSMA PET

**FIGURE 4.**
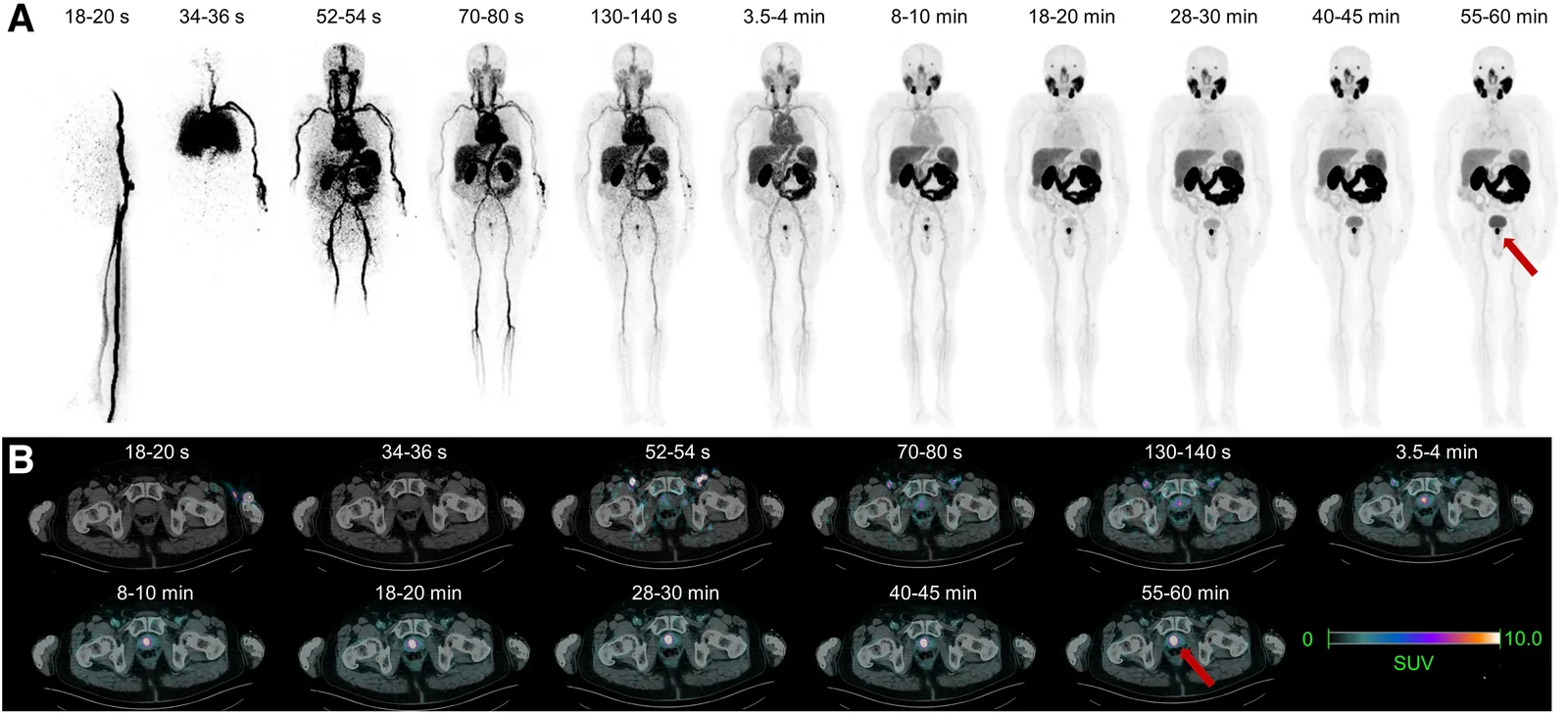
Dynamic total-body PSMA PET. 76-y-old patient with PCa (PI-RADS 2; initial PSA, 19.7; PSA density, 0.82). Representative reconstructed dynamic total-body PET maximum-intensity projection images (A) and transaxial fused PET/CT images (B). Red arrows indicate area of intense uptake in prostate (PRIMARY score 5; ISUP 4 after biopsy; SUV_max_, 55.6).

Among the 11 men who underwent 60-min dynamic total-body PET, 7 of the 11 (64%) had csPCa (ISUP ≥2) and 9 of the 11 (82%) showed positive intraprostatic uptake (PRIMARY ≥3) (Fig. 4). Standard SUV imaging identified 21 lesions (Supplemental Table 4), of which 10 of 17 (59%) with a PRIMARY score of 3 or greater were confirmed csPCa (PET-positive/csPCa-positive, i.e., true positive) whereas 7 of 17 lesions were PET-positive/csPCa-negative, corresponding to false-positive findings. Lesions harboring csPCa (true positive) showed rapid and continuous tracer accumulation, whereas PET-positive/csPCa-negative (false positive) and benign areas displayed an early peak followed by a plateau (Supplemental Fig. 4).

**FIGURE 5.**
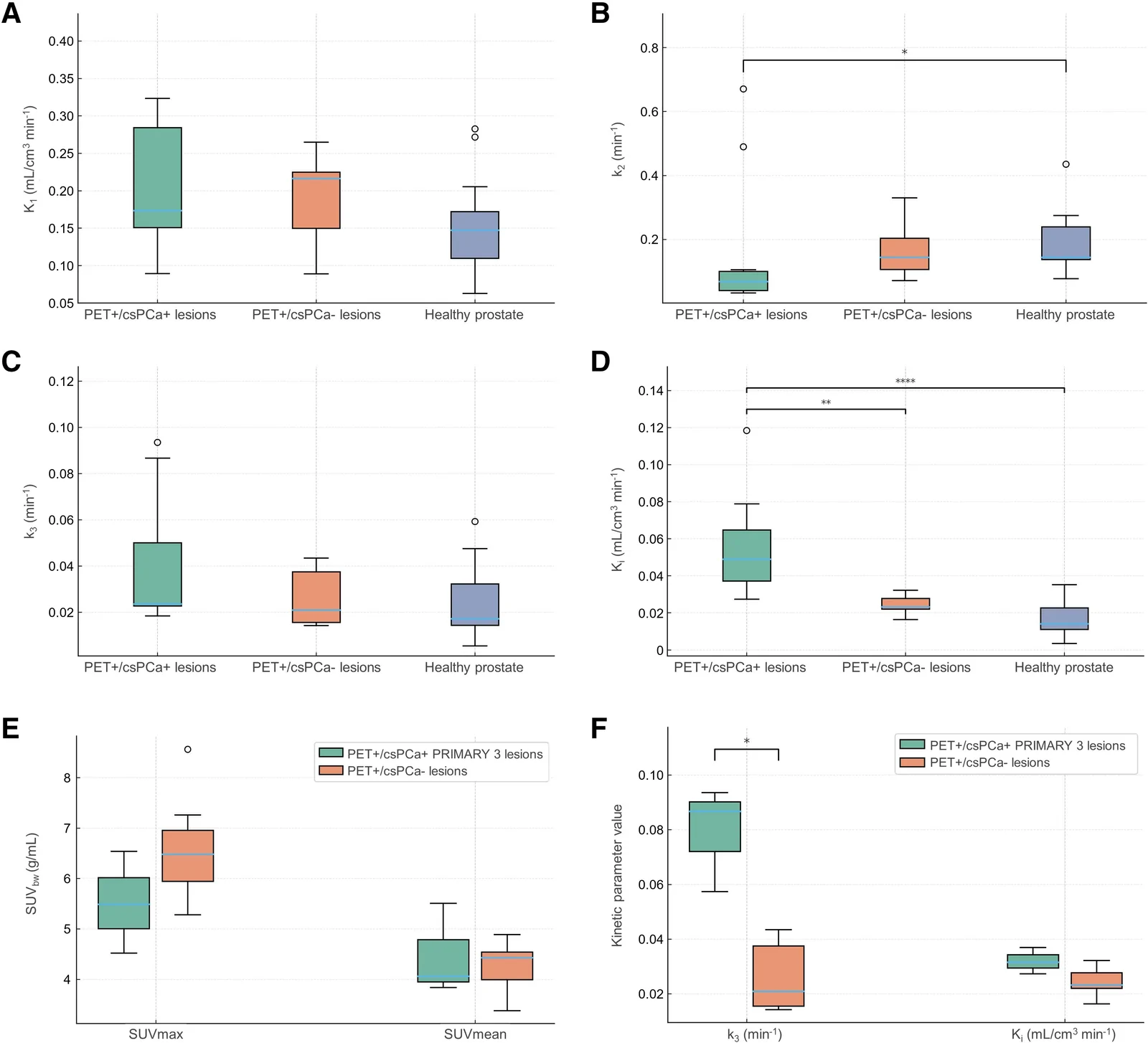
Box plot of kinetic parameters including *K*_1_ (A), *k*_2_ (B), *k*_3_ (C and F), *K_i_* (D and F), and SUVs (E) comparing PET-positive/csPCa-positive lesions (green), PET-positive/csPCa-negative lesions (orange), and healthy prostate (blue). Subanalysis of PET-positive/csPCa-positive PRIMARY score 3 lesions vs. PET-positive/csPCa-negative lesions is presented in panels E and F. Boxes show interquartile range with median, whiskers are 1.5× interquartile range, points show outliers, and asterisks indicate significance between groups: *P < 0.05, **P < 0.01, ***P < 0.001.

Multitime graphical analysis modeling (Patlak and relative equilibrium analysis) revealed that all lesions with irreversible uptake corresponded to csPCa at histology (Supplemental Fig. 5). Accordingly, the irreversible 2-tissue compartment model was preferred and demonstrated higher *K*_1_, *k*_3_, and *K_i_* values and lower *k*_2_ in csPCa compared with reference prostate data (Fig. 5; Supplemental Table 5). Also, *K_i_* was significantly higher in csPCa than in false-positive (PET-positive/csPCa-negative) lesions (*P* = 0.0018), correlated strongly with SUV_max_ (ρ = 0.94, *P* < 0.0001) (Supplemental Table 6), and outperformed SUV_max_ in receiver-operating-characteristic curve analysis (AUC, 0.97 vs. 0.92) (Supplemental Fig. 6). In a PRIMARY 3 versus false-positive (PET-positive/csPCa-negative) lesions subanalysis, *k*_3_ discriminated against the groups (*P* = 0.0167), whereas SUVs did not (*P* > 0.05).

## DISCUSSION

In this prospective single-center study, we demonstrated that PSMA PET-TB significantly outperformed SB in men with negative or equivocal mpMRI. Although SB alone detected csPCa in 21% of patients, PSMA PET-TB identified nearly twice as many cases (37%), achieving a sensitivity of 96% and an NPV of 95%. When combined, the 2 approaches increased the detection rate up to 41%. The poor agreement observed between PSMA PET-TB and SB suggests that each approach identifies a partially different subset of csPCa, supporting the need to combine both techniques to maximize diagnostic yield. Our findings suggest that PSMA PET can refine risk stratification and guide biopsy in a diagnostically challenging and highly selected population (i.e., with negative or equivocal findings), where reliance on mpMRI alone may lead to either underdiagnosis or unnecessary repeated biopsy procedures.

Our results align with and extend previous reports on the added value of PSMA PET in primary diagnosis (24). The PRIMARY study, which enrolled men across the full PI-RADS 2–5 spectrum, showed a sensitivity of 90% and an NPV of 80% for PSMA PET, whereas the combination of PSMA with mpMRI reached a sensitivity of 97% and an NPV of 91% (5). In our more-selective cohort of PI-RADS 2–3 patients only, PSMA-targeted biopsy reached comparable performance (96% sensitivity and 95% NPV), despite the greater diagnostic challenge. Moreover, both studies highlighted the discriminatory power of SUV_max_: in the PRIMARY study, a cutoff of 12.0 conferred 100% specificity for csPCa, whereas in our cohort, an almost identical threshold of 11.4 was observed. These concordant findings reinforce SUV_max_ as a robust quantitative biomarker that correlates with histologic aggressiveness and may support patient selection for targeted biopsy.

Of note, we enrolled a heterogeneous group of men, with approximately one third having undergone a prior biopsy before study inclusion, whereas the PRIMARY study excluded men with a history of prostate biopsy. In our cohort, biopsy-naïve patients had a higher rate of csPCa detection compared with those undergoing repeat biopsy or active surveillance (48% vs. 30%).

The ongoing multicenter, phase 3 PRIMARY 2 study (NCT05154162) randomizes men with PI-RADS 2–3 to PSMA PET/CT–guided management versus template biopsy, with coprimary endpoints of csPCa detection noninferiority and biopsy avoidance; its results will clarify whether PSMA PET can safely reduce biopsies in the PI-RADS 2–3 setting (25).

Our observations are also in line with a systematic review and meta-analysis that reported pooled sensitivity and NPV of 89% and 78% for PSMA PET–targeted biopsy, respectively, and emphasized its particular value in PI-RADS 3 lesions (6). More recently, a comprehensive meta-analysis reported pooled sensitivity and specificity of 82% and 67%, respectively, for intraprostatic csPCa detection, with accuracy improving when PSMA PET was combined with MRI (26). Importantly, the authors caution that NPV alone is insufficient to omit biopsy, reinforcing our pragmatic stance that PET should complement, not wholesale replace, template sampling, whereas its integration with mpMRI and real-time fusion targeting offers the greatest diagnostic leverage.

A further novel aspect of our study is the demonstration of the feasibility and efficacy of real-time PSMA-guided fusion targeted biopsy. In PRIMARY, targeted sampling was performed using static key images provided to the urologist, effectively reflecting a “cognitive” approach (5). By contrast, our platform integrated PSMA PET–defined regions of interest directly into a real-time fusion system with transrectal ultrasound, enabling precise sampling of suspicious foci. This workflow was associated with a higher csPCa detection rate and improved precision and reproducibility compared with cognitive targeting. These results corroborate the recent pilot study by Bianchi et al. which first demonstrated the technical feasibility and strong diagnostic performance of real-time fusion PSMA-targeted biopsy in MRI-negative men (22). In that study, fusion PSMA-TB alone detected over 50% of csPCa cases, with only marginal added yield from SB. Together with our findings, these data provide robust support for the clinical implementation of dedicated fusion platforms, which maximize the diagnostic value of PSMA PET while reducing operator dependence inherent to cognitive targeting.

The exploratory dynamic total-body PET subanalysis offers a novel perspective. Multitime graphical analysis showed a shift from reversible to irreversible PSMA kinetics from healthy prostate to csPCa, with every irreversibly fitted area corresponding to csPCa on histology. Two-tissue modeling corroborated this pattern: biopsy-proven lesions exhibited higher *K*_1_ and *k*_3_ with lower *k*_2_ versus healthy tissue, consistent with increased PSMA binding/internalization and reduced efflux. Although limited by sample size, *K_i_* and *k*_3_ improved discrimination between true- and false-positive PET findings, particularly in PRIMARY score 3 lesions, where uncertainty is greatest and csPCa was detected in only 33%. These results are preliminary and require validation in larger cohorts, but they illustrate the potential of total-body dynamic PSMA PET to refine lesion characterization and generate multiparametric biomarkers beyond static SUV. In our view, these proof-of-concept data justify future multicenter studies rather than immediate clinical adoption.

Several limitations should be acknowledged. First, this was a single-center study with a relatively small cohort and an intentionally enriched population (inclusion criteria for a high-risk subgroup), resulting in a higher prevalence of csPCa than typically reported in unselected PI-RADS 2–3 populations. Accordingly, these findings should not be extrapolated to population-level screening. Second, SB was performed using a limited-core transperineal approach rather than saturation templates. Although this may reduce the sensitivity of systematic sampling, it reflects contemporary clinical practice and highlights the inherent limitations of histologic reference standards based on random sampling. Third, although PSMA PET achieved high sensitivity, specificity was modest (49%). This may raise concerns about false positives; however, nearly all higher-grade cases were correctly identified, underscoring the high sensitivity of PSMA PET for clinically relevant disease.

The relatively long interval between mpMRI and biopsy may have introduced potential misclassification due to disease evolution, although PSA values remained overall stable, partially mitigating this concern.

Lastly, the dynamic PET analysis was exploratory and included few patients. We recognize this limitation but present it as an early signal that kinetic profiling may enhance diagnostic performance in equivocal cases.

Taken together, our results suggest that PSMA PET/CT, especially when integrated with targeted biopsy platforms, can substantially improve the diagnostic yield in men with negative or equivocal mpMRI. Quantitative SUV_max_ and exploratory kinetic parameters from total-body PET further refine lesion assessment and may represent the next frontier in PCa imaging. Validation in larger multicenter trials will be essential, but our study supports the inclusion of PSMA PET into future diagnostic algorithms, with the potential to reduce missed csPCa and avoid unnecessary biopsies in patients at diagnostic uncertainty.

## CONCLUSION

PSMA PET-TB improves detection of csPCa in men with negative or equivocal mpMRI, outperforming SB and adding independent value through quantitative parameters such as SUV_max_. PET should complement rather than replace template sampling, while integration with mpMRI and fusion targeting provides maximal diagnostic yield. Preliminary dynamic total-body PET data suggest that kinetic modeling may further refine lesion characterization, particularly in equivocal cases. These results support the inclusion of PSMA PET in diagnostic pathways for this challenging cohort.

## FUNDING

The work reported in this publication was funded by the Italian Ministry of Health, RC-2026-2801360.

## DISCLOSURE

Stefano Fanti and Andrea Farolfi have received travel and accommodation support and honoraria for educational lectures from United Imaging Healthcare. No other potential conflict of interest relevant to this article was reported.

## Data Availability

Data are available at https://doi.org/10.5281/zenodo.20019909.

## Notes

KEY POINTS **QUESTION:** Can PSMA PET/CT–guided targeted biopsy and semiquantitative PET parameters improve detection of csPCa in men with negative or equivocal mpMRI? **PERTINENT FINDINGS:** In this prospective cohort study of 63 men with PI-RADS 2–3 mpMRI findings, PSMA PET/CT–guided targeted biopsy detected more csPCa than SB alone (37% vs. 21%), with high sensitivity and NPV. SUV_max_ was independently associated with clinically significant disease, and exploratory dynamic total-body PET demonstrated distinct kinetic patterns in aggressive lesions, particularly in equivocal cases. **IMPLICATIONS FOR PATIENT CARE:** PSMA PET/CT may complement mpMRI and SB in diagnostically unresolved patients by improving lesion targeting and providing quantitative biological information that supports more informed biopsy decisions.
